# Keystone Veterinary Medical Association

**Published:** 1901-11

**Authors:** E. M. Ranck

**Affiliations:** Secretary


Secretary.

### KEYSTONE VETERINARY MEDICAL ASSOCIATION.

THE regular monthly meeting of this Assocation was held at the regular meeting-place, northwest corner of Broad and Filbert Streets, on October 8th. Dr. Carter, acting as Chairman, called the meeting to order at nine o'clock, the following members of the profession being present: Drs. Schreiber, Marshall, Adams, Harger, Lintz, Huidekoper, Carter, Mahaffey, Hoskins, Rhoads, Eves, Gardiner, and Ranck.

The minutes of the previous meeting were read and approved. There being no regular papers prepared, Dr. Harger made an elegant address on "Raw, Pasteurized, and Cooked Milk," taken from *Bulletin No* 77 *of the Maryland Agricultural Station*, published August, 1901, compiled by C. F. Doane and T. M. Price, the conclusions of which were:

1. Raw milk is more easily digested when fed to calves than either Pasteurized or cooked milk.

2. Contrary to theory, cooked milk when fed to calves used in these experiments caused violent scouring in the majority of the trials.

3. A majority of physicians in charge of children's hospitals corresponded with favored the use of raw mik for infants when the milk is known to be in perfect condition, but favored Pasteurized milk under ordinary conditions.

4. With one exception, all the physicians corresponded with discourage the use of cooked or sterilized milk for infant feeding.

5. Skim milk was found to be as digestible as whole raw milk.

This valuable talk was freely discussed by Drs. Adams, Hoskins, Schreiber, Lintz, Eves, and others.

An interesting question was proposed by Dr. Hoskins in relation to a condition existing on a valuable dairy farm in which contagious abortion has appeared, and the puzzling part of it is, How are we to meet these conditions when found? The question brought forth many suggestions from the members present. Next in order was the lunch, which, fortunately, did not menace the literary part of the programme.

Election of officers came next, with the following result: Dr. W. L. Rhoads, President; Dr. J. W. Adams, Vice-President; Dr. E. M. Ranck, Secretary; Dr. C. J. Marshall, Treasurer. Directors: Dr. W. Horace Hoskins, Dr. H. P. Eves, Dr. A. P. Schreiber, Dr. B. M. Underhill, Dr. J. D. Houldsworth.

Promises were given for the preparation of papers from Drs. Adams and Harger for the November meeting. A general experience meeting followed, in which everyone present promised to do the best he could to make this season's meetings successful, after which the meeting adjourned at 11 P.M.

E. M. RANCK,
Secretary.

